# LMNB2 promotes the progression of colorectal cancer by silencing p21 expression

**DOI:** 10.1038/s41419-021-03602-1

**Published:** 2021-03-29

**Authors:** Chen-Hua Dong, Tao Jiang, Hang Yin, Hu Song, Yi Zhang, Hao Geng, Pei-Cong Shi, Yi-Xin Xu, Hong Gao, Lian-Yu Liu, Lei Zhou, Zhao-Hui Zhang, Jun Song

**Affiliations:** 1 Department of General Surgery, The Affiliated Hospital of Xuzhou Medical University, Jiangsu 221002 Xuzhou, China; 2 The Graduate School, Xuzhou Medical University, Xuzhou, Jiangsu China; 3 Institute of Digestive Diseases, Xuzhou Medical University, Xuzhou, Jiangsu China; 4 General Surgery, 97th Hospital of Chinese People’s Liberation Army, Xuzhou, 221004 China

**Keywords:** Cancer, Diseases

## Abstract

Colorectal cancer is the second common cause of death worldwide. Lamin B2 (LMNB2) is involved in chromatin remodeling and the rupture and reorganization of nuclear membrane during mitosis, which is necessary for eukaryotic cell proliferation. However, the role of LMNB2 in colorectal cancer (CRC) is poorly understood. This study explored the biological functions of LMNB2 in the progression of colorectal cancer and explored the possible molecular mechanisms. We found that LMNB2 was significantly upregulated in primary colorectal cancer tissues and cell lines, compared with paired non-cancerous tissues and normal colorectal epithelium. The high expression of LMNB2 in colorectal cancer tissues is significantly related to the clinicopathological characteristics of the patients and the shorter overall and disease-free cumulative survival. Functional analysis, including CCK8 cell proliferation test, EdU proliferation test, colony formation analysis, nude mouse xenograft, cell cycle, and apoptosis analysis showed that LMNB2 significantly promotes cell proliferation by promoting cell cycle progression in vivo and in vitro. In addition, gene set enrichment analysis, luciferase report analysis, and CHIP analysis showed that LMNB2 promotes cell proliferation by regulating the p21 promoter, whereas LMNB2 has no effect on cell apoptosis. In summary, these findings not only indicate that LMNB2 promotes the proliferation of colorectal cancer by regulating p21-mediated cell cycle progression, but also suggest the potential value of LMNB2 as a clinical prognostic marker and molecular therapy target.

## Introduction

Colorectal cancer (CRC) is one of the most common malignant tumors worldwide. According to the latest data, the incidence of CRC ranks third in the incidence of malignant tumors and the mortality rate ranks second^1^. In recent years, due to early detection and advanced treatment, CRC mortality has declined^2^. However, the complex developmental mechanism of CRC still hinders the treatment of this disease. Therefore, more detailed mechanisms and reliable markers are needed to predict the survival of CRC patients.

Lamins are components of the nuclear lamina, a fibrous layer on the nucleoplasmic side of the inner nuclear membrane, which is thought to provide a framework for the nuclear envelope^3^. The C terminus is composed of a highly conserved layer tail domain and is involved in protein–protein interactions and protein–nucleus interactions^4–7^. The lamins also have other functions, such as playing a role in chromatin organization, connecting the nucleus and cytoplasm, regulating gene transcription, and mitosis^8–10^. Studies have shown that lamin A also regulates gene expression by binding chromatin to the periphery of the nucleus^11^. Lamin A/C is involved in gene expression related to cell cycle regulation^12^. Lamin A/C is overexpressed in neuroblastoma^13^, prostate cancer^14^, hepatocellular carcinoma^15^, breast cancer^16^, and low-grade endometrial cancer^17^.

The human *LMNB2* gene is located on chromosome 19p13.3^18^ and encodes a 68 kDa protein. Its main function is to maintain the integrity of the nuclear skeleton and participate in cell proliferation and ageing, gene expression, and DNA damage repair by affecting chromosome distribution^19,20^. Lamin B2 is overexpressed in hepatocellular carcinoma^21^. Mutations in the *LMNB2* gene cause progressive myoclonic epilepsy with early ataxia^19,22^. Lamin B2 is overexpressed in ovarian cancer. In addition, LMNB2 can promote the proliferation and tumor formation of non-small cell lung cancer^23,24^.

This study reported for the first time the effect of LMNB2 on the proliferation of CRC cells and explained the molecular mechanism of LMNB2 in the proliferation of CRC. These data provide new insights into the pathogenesis of CRC and support the potential value of LMNB2 as a treatment target for this disease.

## Materials and methods

### Patient and specimens and immunohistochemistry

All CRC patient specimens were obtained from the Pathology Department of Affiliated Hospital of Xuzhou Medical University. The clinicopathologic information of the patients was obtained from their hospital medical records and informed consent was obtained from all patients. The use of these specimens and data for research purposes was approved by the Ethics Committee of the Hospital. Immunohistochemistry (IHC) assays and scoring standards for LMNB2 staining intensity were performed as perviously described^25^. The tissue microarray (TMA) slide was incubated with monoclonal rabbit anti-LMNB2 (1:100, ab151735, Abcam, Cambridge, MA, USA) overnight at 4 °C, anti-p21 antibodies were applied at 1:100 dilutions (#2947, Cell Signaling Technology, USA), and anti-Ki67 antibodies were applied at 1:200 dilutions (ab16667, Abcam, USA). The immunoreactivity was assessed blindly by two independent observers using light microscopy (Olympus BX-51) and the image was collected by a Camedia Master C-3040 digital camera (Beijing, China).

### Cell lines and cell culture

The CRC cell lines FHC, HCT116, DLD1, LOVO, HCT29, SW480, and SW620 were purchased from the Shanghai Institute of Biochemistry and Cell Biology, Chinese Academy of Sciences (Shanghai, China). HCT116, SW480, and SW620 cells were cultured in Dulbecco’s modified Eagle’s medium, whereas DLD1 and LoVo cells were cultured in RPMI 1640 medium supplemented with 10% fetal bovine serum and were incubated in a 37 °C humidified incubator with 5% CO_2_.

### Small interfering RNA and transient transfections

Small interfering RNA (siRNA) specific for LMNB2 (siLMNB2) and nonspecific control siRNA (siCtrl) were purchased from GenePharma (Shanghai, China), and were transfected with siLentFect Lipid Reagent (Bio-Rad Laboratories, Inc.) according to the manufacturer’s protocol, when CRC cells were grown to ~50% confluency. Six hours after transfection, the medium containing transfection reagents was replaced by fresh medium. The siRNA sequences are listed in Supplementary Table S1.

### Stable cell line generation

For stable suppression of LMNB2 expression, LMNB2 short hairpin RNA (shRNA) and control lentivirus were obtained from GenePharma. HCT116 and DLD1 cells were infected with lentivirus for 48 h and then selected with 2 ng/ml puromycin for 2 weeks, with the medium refreshed every 3 days. The shRNA target sequences are listed in Supplementary Table S1.

### Cell proliferation and colony formation assays

Cell proliferation was assessed using CCK8 kit (Bio-Rad Laboratories, Inc.) according to the manufacturer’s instructions. Cells (2 × 10^3^) suspended in 200 μl medium were seeded in triplicate in a 96-well plate, grown at 37 °C. Medium containing 10% CCK8 replaced the original medium and incubated at 37 °C for 2 h, and the absorbance was finally determined at 450 nm using a micro plate reader. For colony formation assay, 1 × 10^3^ cells were cultured in 60 mm plate at 37 °C for 14 days, visible colonies were washed twice with phosphate-buffered saline (PBS), fixed, and stained with 4% paraformaldehyde and crystal violet, respectively. The number of colonies was counted visually.

### EdU assay

EdU (5-Ethynyl-2’-deoxyuridine) assay was used an EdU assay kit (RiboBio, Guangzhou, China) according to the manufacturer’s protocol.The cells were cultivated in 96‐well plates at 4 × 10^3^ cells/well. Twenty hours after culture, the cells were treated with 50 μmol/L Edu and incubated for 2 h at 37 °C. The cells were fixed with 4% paraformaldehyde for 20 min and then permeabilized with 0.5% Triton X‐100 for another 20 min and incubated with 100 μL of 1× Apollo® reaction cocktail for 30 min at room temperature. Finally, the nuclei of the cells were dyed with 100 μL of Hoechst 33342 (5 μg/mL) for 20 min and visualized with fluorescence microscopy (IX71; Olympus, Tokyo, Japan).

### RNA sequencing

RNA sequencing (RNA-seq) was performed by BGI (Wuhan, China). The samples were six in total and were divided into two groups, one of which was the control group and the other was the LMNB2-knockdown group. DEseq2 algorithm was used to identify differentially expressed genes (DEGs) between control and LMNB2 siRNA-treated samples. To improve the accuracy of DEGs, we defined genes with a fold change ≥ 1.5 and adjusted *P*-value ≤ 0.05 as significant DEGs.

### Cell cycle analysis

Cells were collected and fixed in ethanol at −20 °C overnight. Then, cells were washed with PBS and resuspended in 500 μl of PBS with 0.2% Triton X-100, 10 mM EDTA, 100 μg/ml RNase A, and 50 μg/ml propidium iodide (PI) at room temperature for 30 min. Then, cell suspensions were assessed by flow cytometry (BD, FACSCantoTM II).

### Apoptosis

Apoptosis assays were carried out using the Annexin V–fluorescein isothiocyanate (FITC)/PI apoptosis detection kit (Nanjing KeyGen Biotech, Inc.) according to the manufacturer’s protocol. Briefly, the cells were collected after transfection with siRNA for 48 h, washed twice with PBS, and resuspended in binding buffer. Sequentially, the cells were stained with Annexin V–FITC and PI at room temperature for 15 min and then analyzed by flow cytometry (BD, FACSCantoTM II).

### Western blot analysis

Western blotting analysis was performed as described before^26^. The specific primary antibodies against LMNB2 (ab151735) and Ki67 (ab16667) were purchased from Abcam. Antibodies against p21 (#2947), p27 (#3686), Cyclin E2 (#4132), and CDK2 (#2546) were obtained from Cell Signaling Technology. Antibodies against glyceraldehyde 3-phosphate dehydrogenase (60004-1-Ig) were purchased from Proteintech.

### RNA extraction and quantitative real-time PCR

TRIzol reagent was used for total RNA extraction (Invitrogen) following the manufacturer’s instructions. After RNA purity was measured, a reverse-transcription reaction was performed. The PrimeScript™ RT kit (1 µg RNA) and gDNA Eraser (Vazyme) were used. The primers for the p21 promoter fragment were reported^25^. The primers are listed in Supplementary Table S1.

### Dual-luciferase reporter assays

HCT116 cells were transiently transfected with siLMNB2 and nonspecific siCtrl using siLentFect™ Lipid Reagent for RNAi (Bio-Rad Laboratories, Inc.), p21 promoter plasmid, and *Renilla* luciferase plasmid. Forty-eight hours post transfection, we collected the cells and measured the activities of both firefly luciferase and *Renilla* luciferase according to the dual-luciferase reporter assay system (Promega, Madison, WI, USA). The internal standard for transfection efficiency was normalized to *Renilla* luciferase activity. The p21-luc plasmid (−2400/+11) was a gift from Dr. Baiqu Huang (The Institute of Genetics and Cytology, Northeast Normal University).

### Chromatin immunoprecipitation assay

Chromatin immunoprecipitation (ChIP) assay was carried out as described previously^25^. Anti-LMNB2 or negative control anti-IgG were incubated with ChIP dilution buffer containing the sheared DNA at 4 °C overnight rotationally. Quantitative reverse transcriptase PCR was carried out to exaggerate the genomic region of LMNB2 flanking the possible LMNB2-binding sites.

### In vivo tumor xenograft model

The animal experiments were approved by the Animal Care Committee of Xuzhou Medical University, Xuzhou, China. Female BALB/c nude mice (6–8 weeks old) were obtained from the Beijing Vital River Laboratory Animal Technology Co., Ltd and were maintained under specific pathogen-free conditions. HCT116 cells (5 × 10^6^ cells) with knockdown of LMNB2 and control cells were injected subcutaneously into the flanks of the mice. Tumor volume (*V*) was monitored every 2 days by measuring the long axis (*L*) and the short axis (*W*) with a Vernier caliper and were calculated with the following formula: *V* = (*L* × *W*2)/2. Twenty days later, the mice were killed and the tumors were weighed and processed to detect the expression of LMNB2, p21, and Ki67 by IHC. In animal studies, the sample size is more than three pairs.

### Statistical analysis

All the statistical analyses were performed by SPSS 23.0 statistical software package (SPSS, Inc., Chicago, IL). The Mann–Whitney *U*-test and Kruskal–Wallis test were implemented to evaluate the relationship between LMNB2 expression and clinicopathological parameters. Data are expressed as the means ± SD. Student’s *t*-test was used to assess differences within treatment groups. Differences were considered significant when *P* < 0.05.

## Results

### LMNB2 is upregulated in CRC tissues and cell lines, and functions as an independent prognostic factor for CRC

**Fig. 1:**
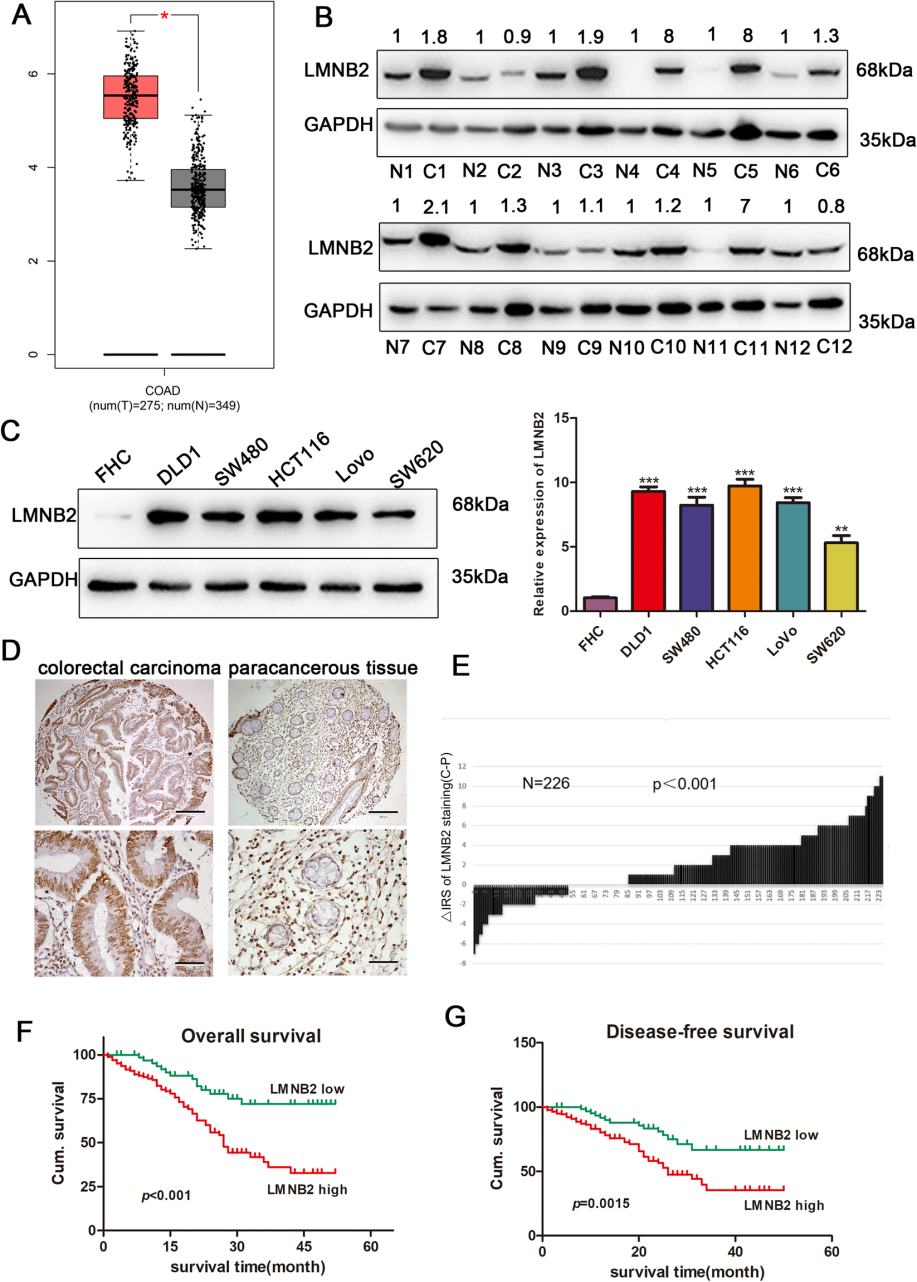
Expression of LMNB2 is upregulated in CRC and negatively associated with overall and disease-free survival in CRC. **A** The GEPIA database shows that LMNB2 is highly expressed in colorectal cancer tissues. **B** Detection of LMNB2 protein levels in 12 cancer tissues and paired normal colon tissues by western blotting. **C** The expression level of LMNB2 was detected by western blotting in five CRC cell lines and a normal colon epithelial cell line (FHC). **D** LMNB2 immunohistochemical staining shows TMA. Note: upper screen, the magnification is ×100; lower screen, the magnification is ×400. **E** Distribution of LMNB2 staining intensity difference between colorectal cancer tissue and paired adjacent tissues. Note: *N* is the paired adjacent non-cancerous tissue. The expression level of LMNB2 in colorectal cancer tissues was significantly higher than that in the corresponding adjacent tissues (paired Wilcoxon’s test, *P* < 0.001). **F** High expression of LMNB2 is associated with poorer overall cumulative survival of patients with colorectal cancer (*P* < 0.001, log-rank test). **G** High expression of LMNB2 is associated with poor disease-free cumulative survival in patients with colorectal cancer (*P* < 0.001, log-rank test).

Gene Expression Profiling Interactive Analysis (GEPIA) analysis found that LMNB2 is differentially expressed in CRC (Fig. 1A). We tested the expression of LMNB2 in 12 pairs of tumor tissues and adjacent tissues, and found that LMNB2 expression was significantly increased in the CRC tissues (Fig. 1B). Western blotting analysis showed that LMNB2 was pervasively highly expressed in CRC cell lines but expressed at relatively low levels in normal colonic epithelial cells (FHC) (Fig. 1C). We analyzed 226 tumor and adjacent tissues, and immunohistochemical staining showed that LMNB2 was mainly located in the nuclear membrane of the nucleus (Fig. 1D). Pairing the tumor tissue with the adjacent tissue and performing a paired Wilcoxon’s test revealed that the LMNB2 protein expression in the cancer tissue was significantly higher than that in the adjacent tissue (*P* < 0.001) (Fig. 1E). Our data showed that CRC patients with high LMNB2 protein expression had worse OS and disease-free cumulative survival (*P* < 0.001) (Fig. 1F, G). We used Fisher’s exact test to investigate the relationship between LMNB2 expression and clinicopathological parameters in patients with CRC. The linicopathological characteristics are summarized in Additional File 2 Table S2. We found that the low expression rate of LMNB2 protein was 28.3% (64/226) and the high expression rate was 71.7% (162/226). The high expression of LMNB2 protein was positively correlated with tumor diameter (*P* < 0.001), TNM stage (*P* < 0.001), lymph node metastasis (*P* = 0.003), distant metastasis (*P* = 0.039), and depth of invasion (*P* = 0.015). In contrast, there was no correlation with age, biological sex, or tumor differentiation (*P* > 0.05). Univariate Cox regression analysis showed that LMNB2 expression, tumor node metastasis (TNM) stage, lymph node metastasis, tumor diameter, and depth of invasion are important prognostic factors affecting overall survival (OS) and disease-free survival (DFS) in patients with CRC (Additoonal File 3 Table S3). In the multivariate Cox regression model, our data further confirmed that the expression of LMNB2 was still associated with the OS of patients with CRC (*P* = 0.007) and DFS (*P* = 0.012) as independent and meaningful prognostic biomarkers (Additoonal File 4 Table S4). In summary, our results confirmed that LMNB2 expression may be a potential independent prognostic factor for OS and DFS in CRC patients.

### LMNB2 promotes the proliferation of CRC cells in vitro

**Fig. 2:**
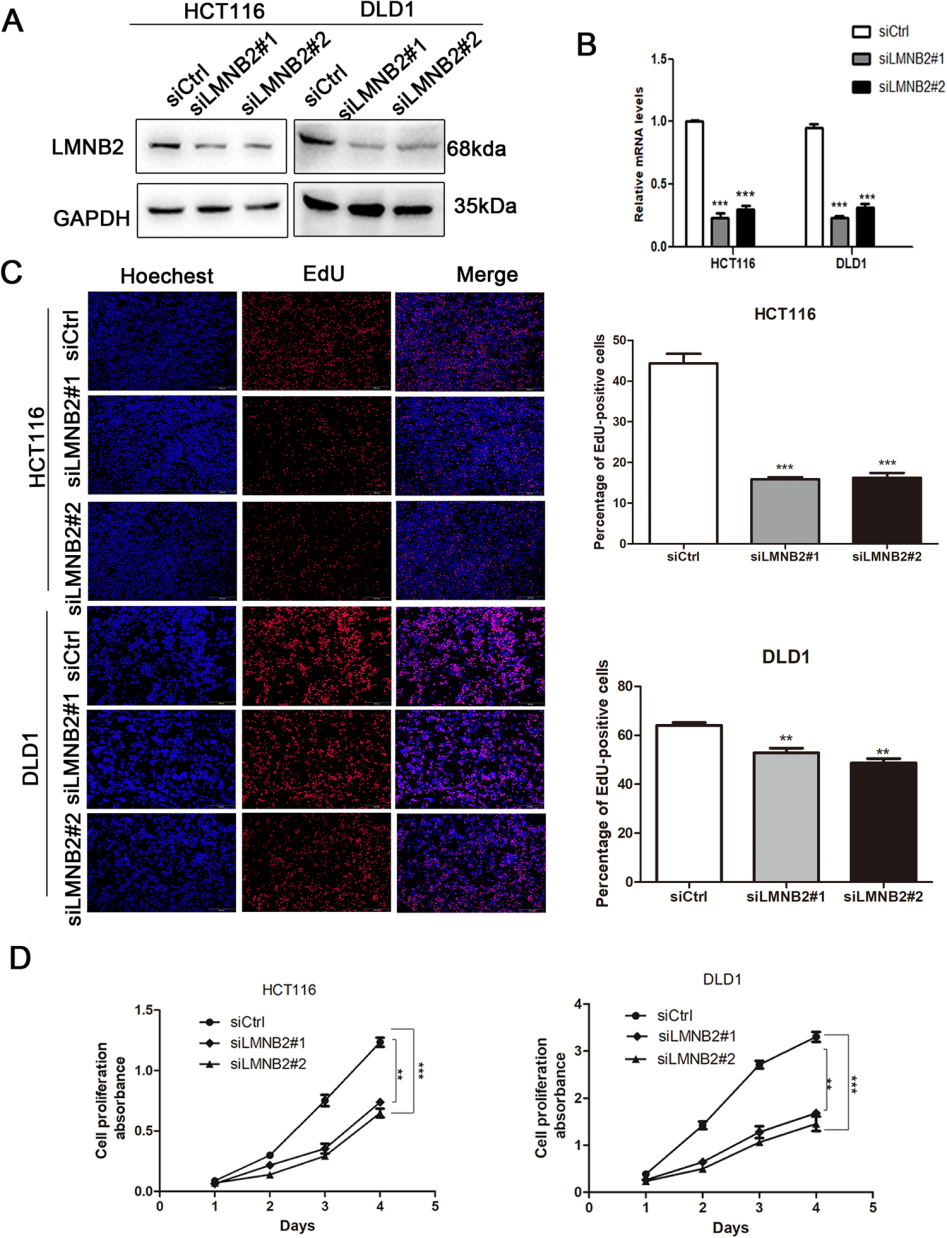
LMNB2 promotes the proliferation of colorectal cancer cells. **A** Western blotting confirmed that the protein level of LMNB2 in HCT116 and DLD1 cells was downregulated. **B** Real-time quantitative PCR confirmed that the mRNA levels of LMNB2 in HCT116 and DLD1 cells were knocked out. **C**, **D** LMNB2 knockout can significantly inhibit the proliferation of HCT116 and DLD1 cells. All experiments are in triplicate. The data are shown as mean ± SD. *P < 0.05, **P < 0.01.

HCT116 and DLD1 cells were transiently transfected with siRNAs targeting LMNB2 (siLMNB2#1 and siLMNB2#2) or siCtrl. The results revealed that LMNB2 expression was significantly reduced in the cells transfected with LMNB2 siRNA compared with the control cells (Fig. 2A, B). The EdU incorporation assay showed that compared with the corresponding control cells, the EdU-positive HCT116 and DLD1 cells in the LMNB2 downregulation group were significantly reduced (Fig. 2C). In CCK8 proliferation assays, the proliferation of HCT116 and DLD1 cells decreased significantly after knocking down LMNB2 expression (Fig. 2D).

### LMNB2 regulates the p21-mediated cell cycle and promotes cell proliferation

**Fig. 3:**
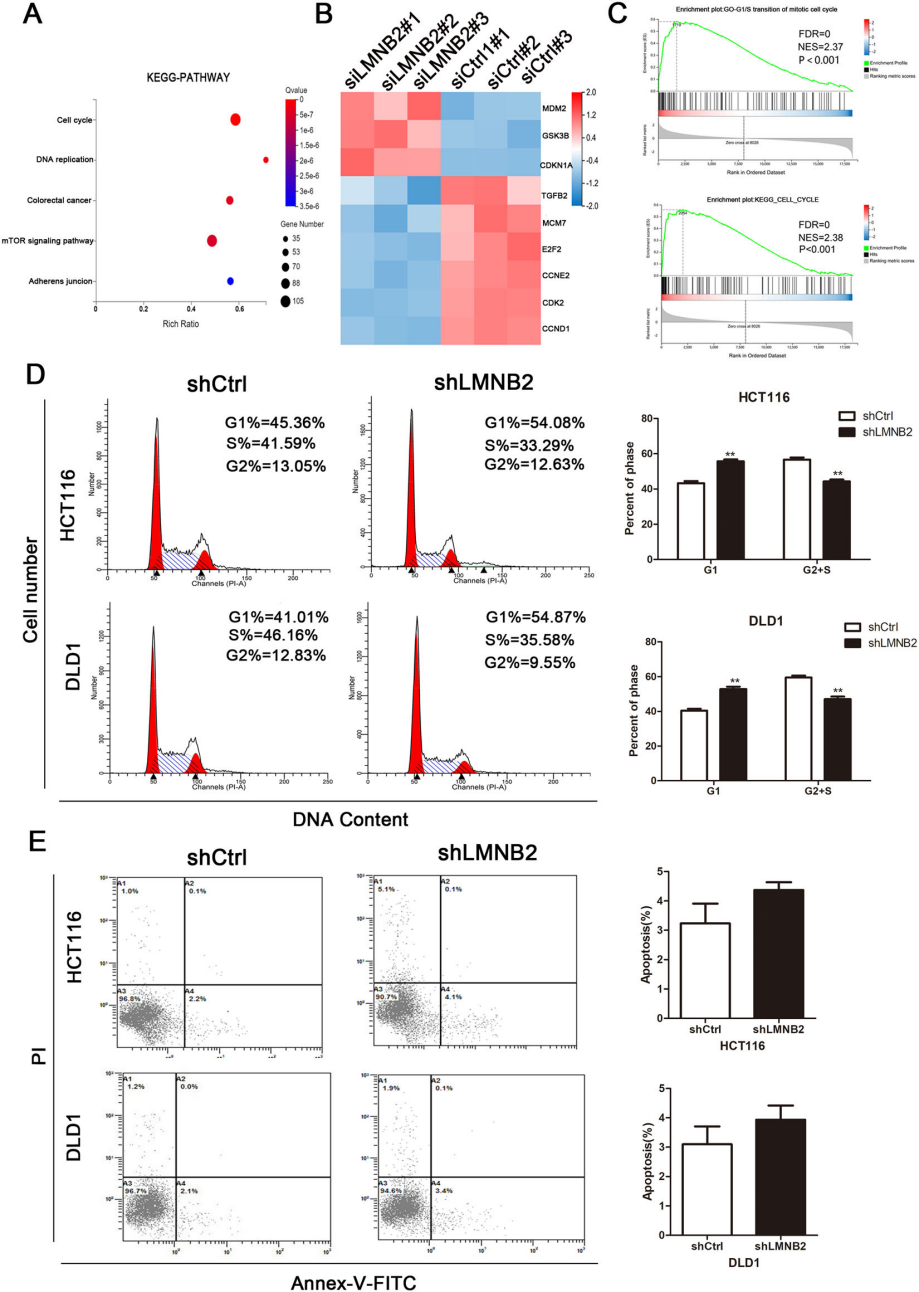
LMNB2 can accelerate the cycle of colorectal cancer cells, but has no effect on cell apoptosis. **A** Kyoto Encyclopedia of Genes and Genomes pathway analysis was performed for screening DEGs using the PANTHER database. **B** Heat map of differentially expressed genes after *LMNB2* gene knockout. **C** GSEA analysis showed that the LPCAT1 amplification status was positively correlated with genes related to “KEGG CELL CYCLE” and “GO CELL CYCLE G1-S TRANSITION”. **D** Flow cytometry analysis after Annexin–FITC and PI staining found that after *LMNB2* gene knockout, the number of cells in G0/G1 phase increased, whereas the number of cells in S phase and G2 phase decreased. **E** Flow cytometry analysis and Annexin PI staining results showed that *LMNB2* gene knockout had no significant effect on the apoptosis of colorectal cancer cells. All experiments were performed independently three times. The data are the mean ± SD. *P < 0.05, **P < 0.01, *P < 0.001.

To identify potential mRNA targets regulated by LMNB2, we performed RNA-seq of DLD1 cells. A total of 7445 genes were identified from mRNA sequencing. These genes are differentially enriched in certain cancer-related pathways. For example, cell cycle pathways showed a strong correlation (Fig. 3A). Among these genes, *p21* (CDKN1A) was significantly upregulated after LMNB2 knockdown (Fig. 3B). In the TCGA database, the expression of p21 in the tissues of CRC patients was less than that in the normal colorectal tissues (Additional File 5 Fig. S1A). GEPIA database shows that LMNB2 is negatively correlated with p21 and positively correlated with CDK2 and Cyclin E2 (Additional File 5 Fig. S1B–D). As GSEA analysis showed that LMNB2 amplification was positively correlated with genes related to “KEGG cell cycle” and “GO cell cycle G1-S transition,” we further studied the effect of LMNB2 on CRC cell cycle regulation (Fig. 3C).

**Fig. 4:**
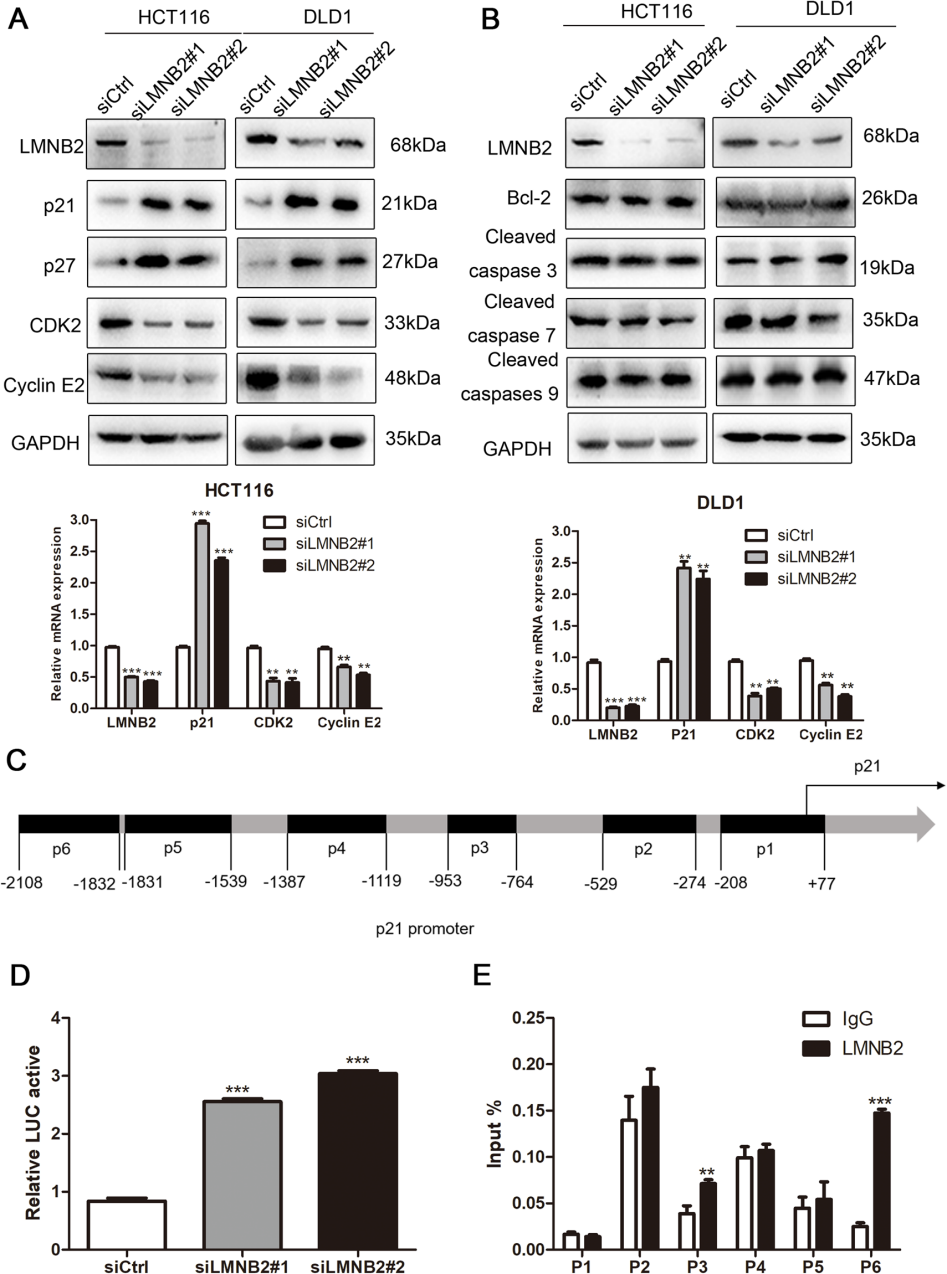
*LMNB2* gene knockout can induce cell cycle arrest in G0/G1 phase and inhibits the transcription of p21. **A** Western blotting was used to detect the expression of cell cycle and apoptosis-related genes. GAPDH was used as a reference control. **B** Real-time fluorescence quantitative PCR detects the relative expression of cell cycle-related genes at the mRNA level. GAPDH was used as a reference control. **C** The complete sequence of the human p21 promoter. P1-6 shows the p21 promoter region detected by two pairs of primers. **D** The activity of p21 promoter (LMNB2 2400/11) in −116 cells was enhanced after *LMNB2* gene knockout. **E** Three-dimensional ChIP quantitative PCR analysis of the binding of LMNB2 at P1, P2, P3, P4, P5, and P6. Western blot detection of LMNB2 in ChIP detection. The mean ± SD of three experiments is given. *P < 0.05, **P < 0.01, *P < 0.001.

Flow cytometry showed that the downregulation of LMNB2 expression resulted in G1 arrest, indicating that LMNB2 is involved in cell cycle regulation (Fig. 3D). However, as apoptosis also has an important effect on cell proliferation, we used flow cytometry to analyze the effect of LMNB2 on cell apoptosis. The results showed that there was no significant change in apoptosis after silencing LMNB2 (Fig. 3E). Western blot analysis showed that knockdown of LMNB2 in the HCT116 and DLD1 cell lines significantly increased the expression of p21. Furthermore, the downregulation of LMNB2 resulted in significant accumulation of p27 protein, whereas it significantly reduced the expression of Cyclin E2 and CDK2. Real-time quantitative PCR confirmed these results and the results showed that LMNB2 negatively regulates p21 at the mRNA level. In addition, the expression of apoptotic factors, including cleaved caspase 3, cleaved caspase 7, cleaved caspase 9, and Bcl-2 did not change significantly (Fig. 4A, B).

### LMNB2 transcriptionally inhibits p21 in CRC cells in vitro

Cell cycle disorders can lead to abnormal cell proliferation and abnormal cell proliferation is closely related to the occurrence of tumors. Any interference with the programs involved in cell cycle regulation may lead to abnormal cell proliferation^27,28^. p21 is an important regulator of the G1/S transition phase of the cell cycle, is considered an important regulator of cell growth inhibition^29,30^, and is involved in regulating G0/G1 phase arrest and cell proliferation^31^. We analyzed the promoter region of the human *p21* gene and cloned it into the luciferase reporter plasmid (Fig. 4C). Subsequently, the dual-luciferase reporter experiment showed that transfection of LMNB2-targeting siRNA significantly increased the activity of the p21 promoter (Fig. 4D), indicating that LMNB2 plays a key role in regulating the expression of p21 in CRC at the transcriptional level. We analyzed the promoter region of the human *p21* gene and designed a potential binding region. These regions are denoted as P1, P2, P3, P4, P5, and P6. To determine whether LMNB2 can be recruited to the potential binding region of the p21 promoter, we performed ChIP analysis in HCT116 LMNB2-knockout cells and control cells. The ChIP quantitative PCR results showed that LMNB2 mainly binds to the P3- and P6-binding regions of the p21 promoter (Fig. 4E).

### LMNB2 promotes the growth of CRC in vivo

**Fig. 5:**
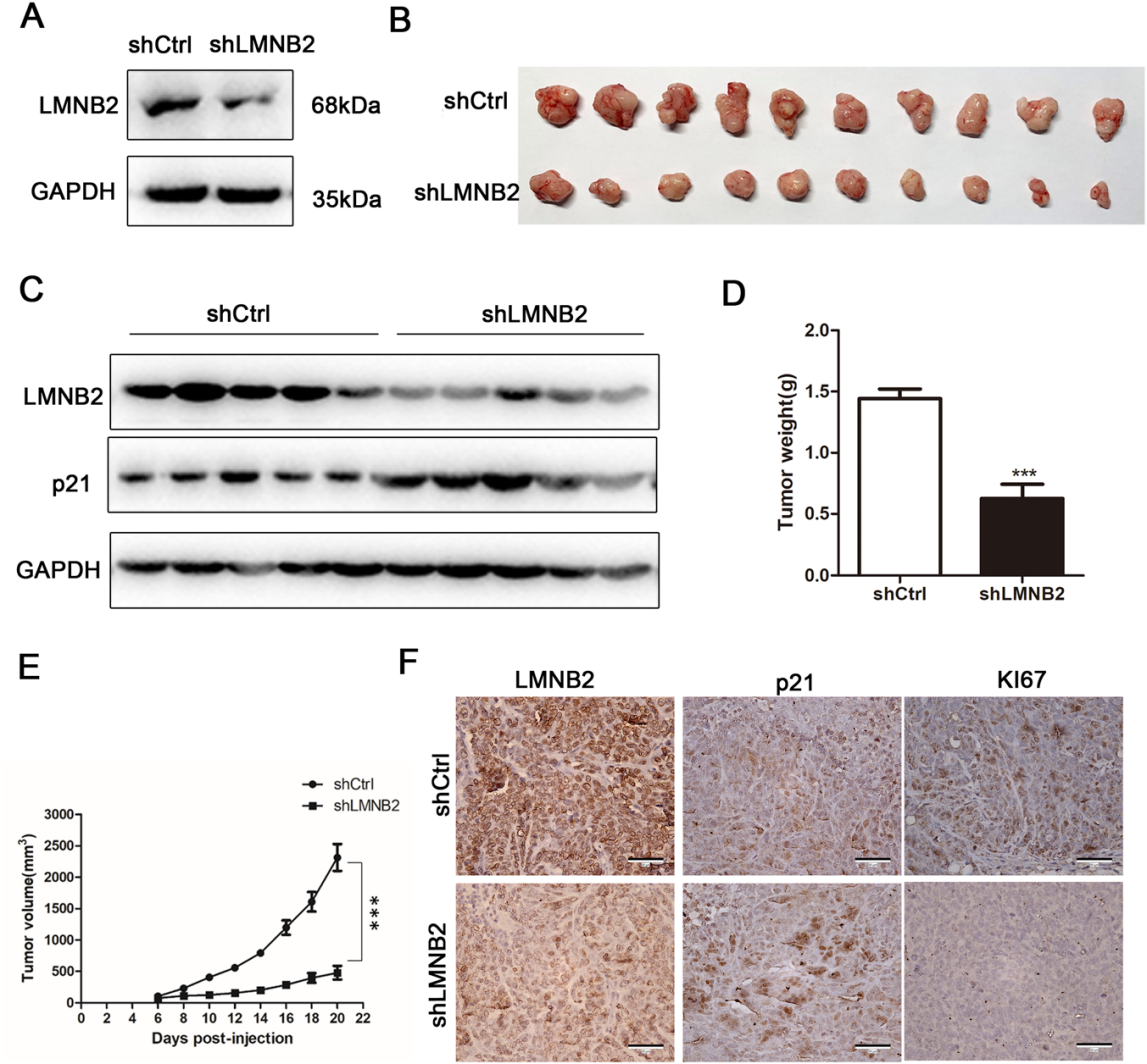
Knockout of the *LMNB2* gene can inhibit tumor formation in colorectal cancer cells. **A** Western blotting confirmed that the *LMNB2* gene was knocked out in the HCT116 stable cell line at the protein level. **B** Gross observation of subcutaneous tumors of nude mice on HCT116 cells transfected with shLMNB2 and shCtrl lentivirus (10 mice per group). The mice were killed 20 days after injection and the transplanted tumors were collected. **C** Detection of LMNB2 protein levels in five cancer tissues and paired normal colon tissues by western blotting. **D** Analyze the weight of the transplanted tumor. **E** The volume of transplanted tumor in nude mice. The calculation formula of transplanted tumor volume is: *V* = (*L* × *W*2)/2. **F** Tumor sections were immunohistochemically stained with antibodies against LMNB2, p21, and Ki67, and a representative image was displayed (magnification: ×400). *P < 0.05. *L* represents the long axis, *W* represents the short axis.

We established a stable cell line in which LMNB2 was knocked down in HCT116 cells in preparation for further research. Western blot analysis showed that LMNB2 was knocked down in a stable cell line (Fig. 5A). We subcutaneously injected the stable cells and the control cells mixed with Matrigel into BALB/c nude mice. After 6 days, we measured the maximum length and width of the subcutaneous tumor every other day. The mice were killed 20 days later and the tumors formed by stable LMNB2-knockout cells were significantly smaller and lighter than those of the control group (Fig. 5B). We randomly selected five pairs of tissues for western blot experiments and found that LMNB2 expression was low in the experimental group and p21 was upregulated in the experiment group (Fig. 5C). The tumor growth and tumor volume of the HCT116 cell group injected with the stable *LMNB2* gene were significantly lower than those of the control group (Fig. 5D, E). In addition, the shLMNB2 and shCtrl tumor tissue sections were subjected to immunohistochemical staining, to detect the expression of LMNB2, p21, and the nuclear proliferation marker Ki67. Representative images showed that knockout of LMNB2 resulted in a decreased staining intensity of LMNB2 and Ki67 in the resected tumor and an increased staining intensity of p21 compared with those of the control group (Fig. 5F).

### Downregulation of p21 reverses the inhibition of proliferation by *LMNB2* gene knockdown

**Fig. 6:**
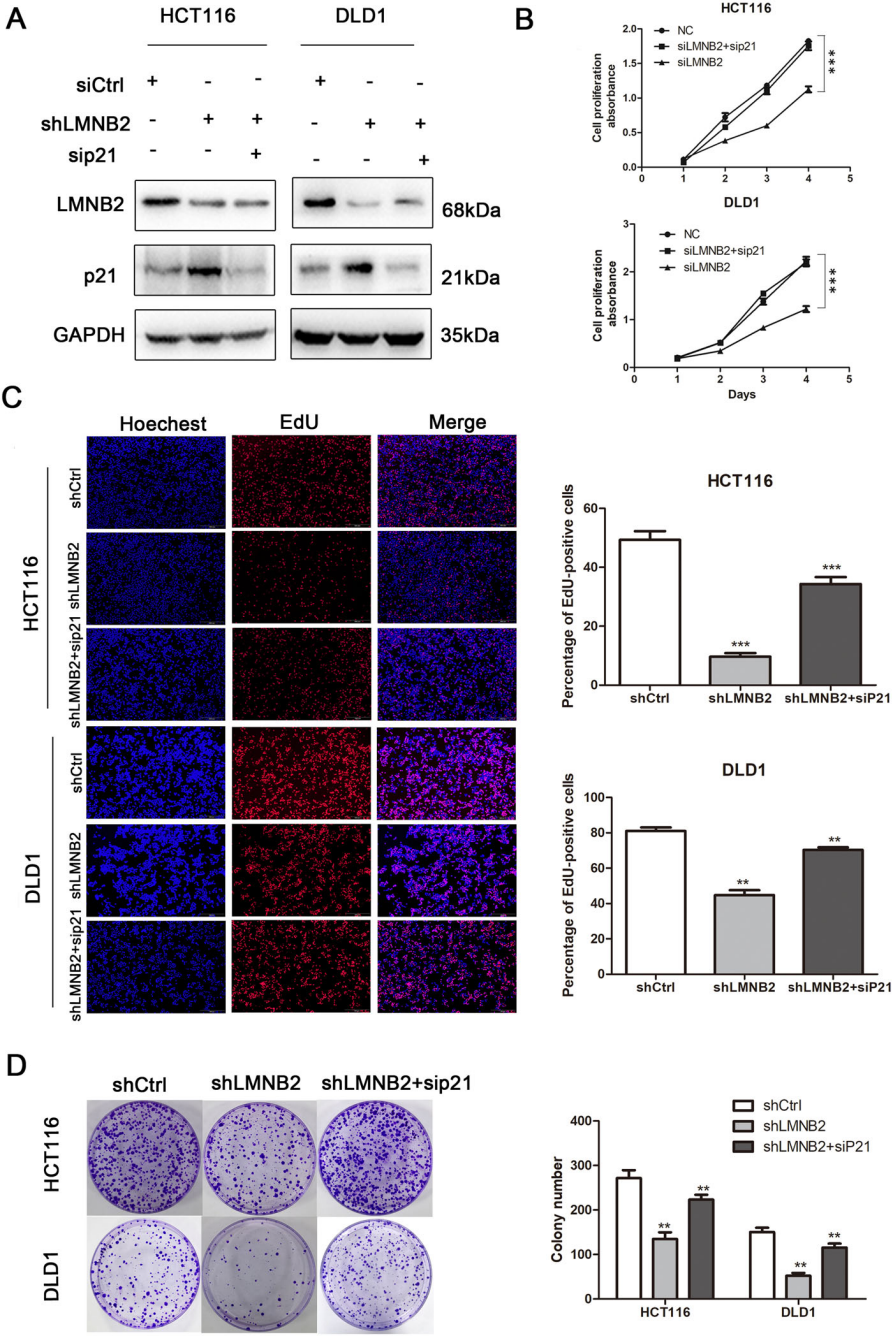
Downregulation of p21 can reverse the inhibition of proliferation caused by *LMNB2* gene knockout. **A** Western blotting confirmed the presence of *LMNB2* and *p21* gene knockout in HCT116 and DLD1 stable cell lines at the protein level. **B**–**D** Transiently transfected with p21 siRNA can reverse the inhibitory effect of *LMNB2* gene knockout on the proliferation of HCT116 and DLD1 cells. The data is shown as the mean ± standard deviation from three independent experiments. *P < 0.05, **P < 0.01, *P < 0.001.

To evaluate the effect of p21 in suppressing the proliferation induced by LMNB2 silencing, we silenced p21 in HCT116 and DLD1 cells with silenced LMNB2 by siRNA (Fig. 6A). The results showed that transiently transfected p21 siRNA can reverse the inhibited proliferation due to *LMNB2* gene knockout in HCT116 and DLD1 stable cell lines (Fig. 6B, C). The clone formation assay showed that the colony-forming ability of the CRC cells transfected with LMNB2 shRNA was weaker than that of the control group, but transiently transfected p21 siRNA can reverse the inhibited proliferation (Fig. 6D). These results indicated that p21 plays an important role in the proliferation of CRC cells regulated by LMNB2.

## Discussion

The occurrence and development of tumors is a multifactor and multistep complex process that includes oncogene activation and tumor suppressor gene inactivation. Therefore, exploring the exact molecular mechanism of CRC progression is important.

Lamins are overexpressed in most cancers and has the ability to maintain cancer cell homeostasis. Lamin B tethering may differentially affect gene expression by forming complexes with different chromatin-binding proteins, epigenetic regulators, and transcription factors in specific chromatin regions^32^. LMNB2 has been shown to play an important role in the occurrence and development of tumors. However, there is no research on the relationship between LMNB2 expression and the progression of CRC.

In this study, the expression of LMNB2 protein in CRC tissues was higher than that in adjacent tissues and the high expression of LMNB2 was related to tumor size, TNM stage, and lymph node metastasis. These findings indicate that LMNB2 plays an important role in the development of CRC and can be used as a potential clinical prognostic indicator for patients with CRC undergoing radical resection. We conducted cell line and animal experiments to explore the possible role of LMNB2 in CRC. Our current data showed that in cultured CRC cells, LMNB2 blockade downregulates the proliferation and colony formation of CRC cells. We performed mRNA sequencing and found that the expression of GSK3B increased after knocking down LMNB2. Studies have shown that GSK3B can inhibit the wnt/β-catenin pathway and thereby inhibit tumor metastasis. Then, whether LMNB2 promotes the metastasis of CRC by regulating the wnt/β-catenin pathway mediated by GSK3B remains to be further studied.

In conclusion, our study shows for the first time that the abnormal expression of LMNB2 promotes the proliferation and development of CRC. LMNB2 promotes cell proliferation of CRC by inducing p21-mediated cell cycle progression. These findings reveal the promise of LMNB2 as a potential biomarker and target for the prognosis and treatment of CRC.

## Supplementary information

Additional file 1 Table S1.

Additional file 2 Table S2.

Additional file 3 Table S3.

Additional file 4 Table S4.

Additional file 5 Figure S1.

## Data Availability

All data generated or analyzed during this study are included in this published article. Further details are available from the corresponding author upon request.
