# Supplementary material for: LMNB2 promotes the progression of colorectal cancer by silencing p21 expression
Source: Cell Death Dis. 2021 Mar 29;12(4):331. doi: 10.1038/s41419-021-03602-1 (PMC8007612; doi:10.1038/s41419-021-03602-1)
Supplement: Supplementary file 1 — Additional file 1 Table S1. [file 41419_2021_3602_MOESM1_ESM.docx]

1. The siRNA sequences are as follows:

SiLMNB2#1 sense: GCGAGGUGAGUGGCAUCAATT;

SiLMNB2#2 sense: GGAAGUGGCCAUGAGGACUTT;

sip21# sense: CCUCUGCAUUAGAAUUAUTT;

siCtrl sense: UUCUCCGAACGUGUCACGUTT.

1. The shRNA target sequences were as follows:

shLMNB2 sense: GCGAGGUUGGCAUCAATT;

shCtrl sense: TTCTCCGAACGTGTCACGT.

1. The primer sequences are as follows:

5′-TGGAGATCAACGCCTACCG -3′ (forward) and

5′-AGCCGCTTCCGCTTACTG -3′ (reverse) for LMNB2;

5′-AAGGTCGGAGTCAACGGATTTG-3′ (forward)

and 5′-CCATGGGTGGAATCATATTGGAA-3′ (reverse) for GAPDH；

5′-TTTCTCTCGGCTCCCCATGT-3′ (forward) and
5′-GCTGTATATTCAGCATTGTGGG-3′ (reverse) for p21;

5′-TAGCTGGTCTGGCGAGGTTT-3′ (forward) and
5′-ACAGGTGGCCAACAATTCCT-3′ (reverse) for cyclin E2;

5′-ACCAACGCAGGCGAGGGA-3′ (forward) and 5′-
CCGGCTCCACAAGGAACT-3′ (reverse) for p1;
5′-GGTGTCTAGGTGCTCCAGGT-3′ (forward) and
5′-GCACTCTCCAGGAGGACACA-3′ (reverse) for p2;
5′-GCAGGAGGCAAAAGTCCTGT-3′ (forward) and
5′-GTGGTTGCAGCAGCTTTGTT-3′ (reverse) for p3;
5′-GAAAGAAGCCTGTCCTCCCC-3′ (forward) and
5′-CGCTCCCATCTACCTCACAC-3′ (reverse) for p4;
5′-CGTGGTGGTGGTGAGCTA-3′ (forward) and 5′-
CTGTCTGCACCTTCGCTCCT-3′ (reverse) for p5;
5′-GTAAACCTTAGCCTGTTACTCTGAA-3′ (forward) and

5′-CATTCAATATTTCTTAAGTACCTAC-3′ (reverse) for p6.
