# Supplementary material for: LMNB2 promotes the progression of colorectal cancer by silencing p21 expression
Source: Cell Death Dis. 2021 Mar 29;12(4):331. doi: 10.1038/s41419-021-03602-1 (PMC8007612; doi:10.1038/s41419-021-03602-1)
Supplement: Supplementary file 2 — Additional file 2 Table S2. [file 41419_2021_3602_MOESM2_ESM.docx]

| Variables | cases | LMNB2 expression(n=226 cases) | | P^a^ |
| --- | --- | --- | --- | --- |
|  |  | Low(%) | High(%) |  |
| All patients | 226 | 64(100) | 162(100) |  |
| Age(years) |  |  |  | 0.285 |
| ≤60 | 101(45) | 25(39) | 76(47) |  |
| ＞60 | 125(55) | 39(61) | 86(53) |  |
| Gender |  |  |  | 0.332 |
| Males | 114(55) | 29(45) | 85(52) |  |
| Females | 112(54) | 35(55) | 77(48) |  |
| Tumor diameter |  |  |  | <0.001 |
| ≤5cm | 128(57) | 48(75) | 80(49) |  |
| >5cm | 98(43) | 16(25) | 82(51) |  |
| Lymph node metastasis |  |  |  | 0.003 |
| N0 | 139(62) | 49(90) | 90(49) |  |
| N1/N2/N3 | 87(38) | 15(10) | 72(51) |  |
| TNM stage |  |  |  | <0.001 |
| Ⅰ~Ⅱ | 120(53) | 48(76) | 72(44) |  |
| Ⅲ~Ⅳ | 106(47) | 16(24) | 90(56) |  |
| Differentiation^b^ |  |  |  | 0.198 |
| Poor | 32(14) | 12(19) | 20(12) |  |
| Moderate/high | 189(86) | 50(81) | 139(88) |  |
| Distant metastasis |  |  |  | 0.039 |
| M0 | 207(84) | 63(98) | 144(89) |  |
| M1 | 19(16) | 1(2) | 18(11) |  |
| Depth of invasion |  |  |  | 0.015 |
| T1/T2 | 56(25) | 23(36) | 33(20) |  |
| T3/T4 | 170(75) | 41(64) | 129(80) |  |

^a^ Two-sided Fisher’s exact tests

^b^The type of differentiation of cancer in five patients cannot be assessed
