# Supplementary material for: LMNB2 promotes the progression of colorectal cancer by silencing p21 expression
Source: Cell Death Dis. 2021 Mar 29;12(4):331. doi: 10.1038/s41419-021-03602-1 (PMC8007612; doi:10.1038/s41419-021-03602-1)
Supplement: Supplementary file 3 — Additional file 3 Table S3. [file 41419_2021_3602_MOESM3_ESM.docx]

| **Variables**^a^ | **Overall survival** | | **Disease-free survival** | |
| --- | --- | --- | --- | --- |
|  | **HR（95% CI）** | **P** | **HR（95% CI）** | **P** |
| LMNB2 | 2.834（1.572-5.109） | 0.001 | 2.668（1.482-4.803） | 0.001 |
| Age | 1.522（0.517-1.384） | 0.505 | 0.830（0.507-1.359） | 0.459 |
| Gender | 0.955（0.577-1.580) | 0.856 | 0.945（0.571-1.563） | 0.824 |
| LNM | 1.687（1.043-2.728） | 0.033 | 1.682（1.040-2.720） | 0.034 |
| Distant metastasis | 1.130（0.410-3.110） | 0.814 | 0.914（0.333-2.511） | 0.862 |
| TNM stage | 2.810（1.712-4.610） | <0.001 | 2.367（1.456-3.847） | 0.001 |
| Differentiate | 1.090（0.556-2.137） | 0.803 | 1.110（0.566-2.176） | 0.762 |
| Tumor Diameter | 2.426（1.418-3.937） | <0.001 | 2.069（1.272-3.363） | 0.003 |
| Depth of invasion | 1.783（1.078-2.949） | 0.024 | 1.985（1.199-3.287） | 0.008 |

HR hazard ratio, CI confidence interval, LNM lymph node metastasis

^a^LMNB2: low vs high; age: ≤60 vs >60; gender: male vs female; LNM: N0 vs N1, N2,

N3; depth of invasion: T1–T2 vs T3–T4; distant metastasis: M0 vs M1;

differentiate: poor vs moderate and high; TNM stage was ranked as I–II vs

III–IV; tumor diameter: ≤5 v s > 5
