# Supplementary material for: LMNB2 promotes the progression of colorectal cancer by silencing p21 expression
Source: Cell Death Dis. 2021 Mar 29;12(4):331. doi: 10.1038/s41419-021-03602-1 (PMC8007612; doi:10.1038/s41419-021-03602-1)
Supplement: Supplementary file 4 — Additional file 4 Table S4. [file 41419_2021_3602_MOESM4_ESM.docx]

| **Variables^a^** | **Overall survival** | | **Disease-free survival** | |
| --- | --- | --- | --- | --- |
|  | **HR（95% CI）** | **P** | **HR（95% CI）** | **P** |
| LMNB2 | 0.429（0.232-0.796） | 0.007 | 0.456（0.248-0.839） | 0.012 |
| Tumor Diameter | 1.765（1.031-3.020） | 0.038 | 1.816（1.085-3.041） | 0.023 |
| TNM stage | 1.762（1.026-3.027） | 0.040 | 1.592（0.943-2.689） | 0.082 |
| LNM | 1.104（0.660-1.847） | 0.707 | 1.161（0.693-1.947） | 0.571 |
| Depth of invasion | 1.617（0.971-2.693） | 0.065 | 1.567（0.923-2.659） | 0.096 |

HR hazard ratio, CI confidence interval, LNM lymph node metastasis

^a^LMNB2: low vs high; age: ≤60 vs >60; LNM: N0 vs N1, N2,

N3; depth of invasion: T1–T2 vs T3–T4;differentiate: poor vs moderate and high;

TNM stage was ranked as I–II vs III–IV; tumor diameter: ≤5 v s > 5
