# Supplementary figures and images for: LMNB2 promotes the progression of colorectal cancer by silencing p21 expression
Source: Cell Death Dis. 2021 Mar 29;12(4):331. doi: 10.1038/s41419-021-03602-1 (PMC8007612; doi:10.1038/s41419-021-03602-1)

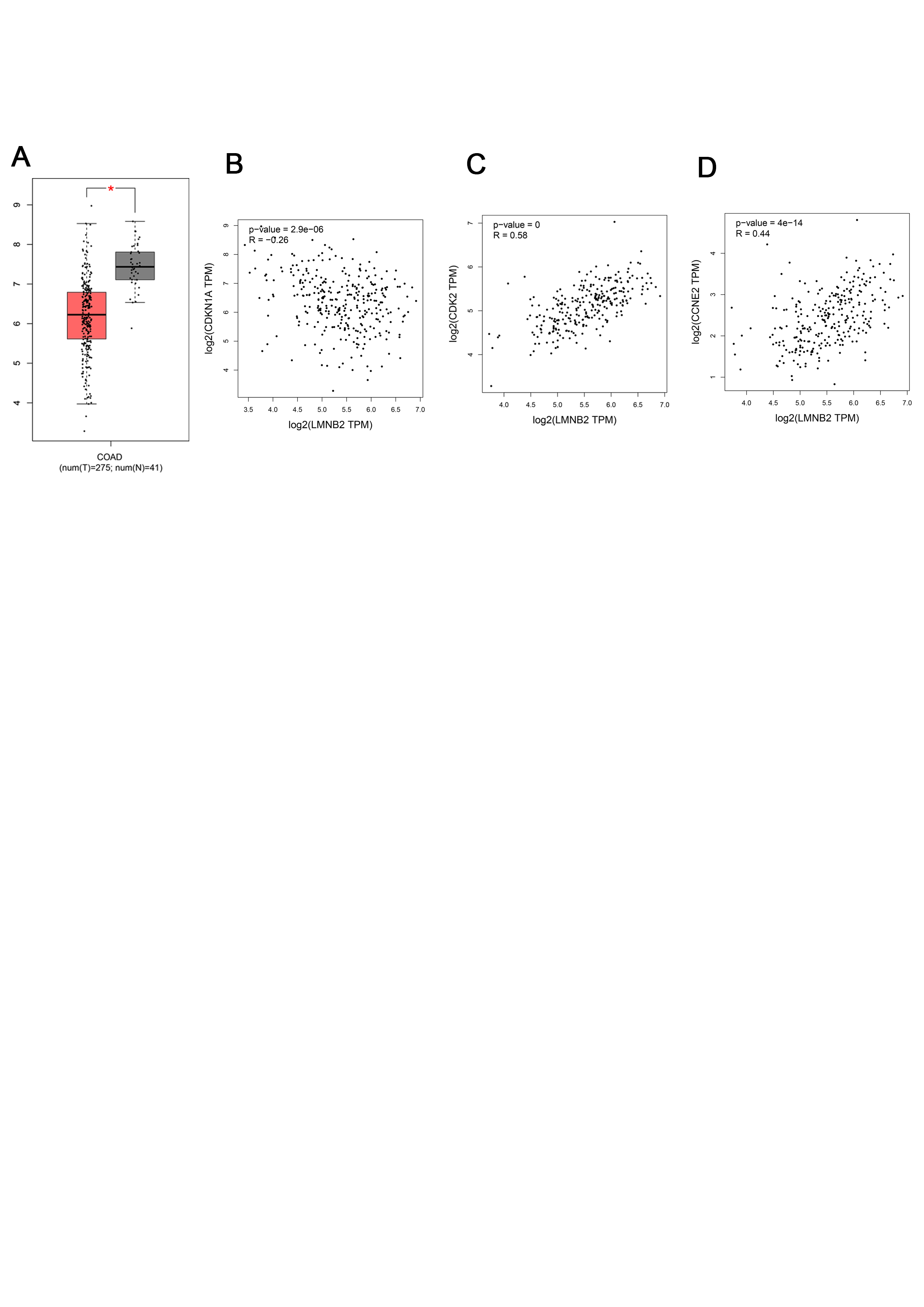

Supplement: Supplementary file 5 — Additional file 5 Figure S1. [file 41419_2021_3602_MOESM5_ESM.tif]
